# RETRACTION: Prediction of Arrhythmia Recurrence after Atrial Fibrillation Ablation in Patients with Normal Anatomy of the Left Atrium

**DOI:** 10.1155/2024/9894257

**Published:** 2024-08-28

**Authors:** International Journal of Clinical Practice

RETRACTION: A. K. Baimbetov, K. A. Bizhanov, K. B. Abzaliyev, B. A. Bairamov, I. A. Yakupova, “Prediction of Arrhythmia Recurrence after Atrial Fibrillation Ablation in Patients with Normal Anatomy of the Left Atrium,” *International Journal of Clinical Practice* 75 (2021): e14083, https://doi.org/10.1111/ijcp.14083.

The above article, published online on 8 February 2021 in Wiley Online Library (https://wileyonlinelibrary.com) [1], has been retracted by agreement between the Chief Editor, Angela Vinturache, and John Wiley & Sons Ltd. UK. The retraction has been agreed following concerns raised by a third party regarding the peer review process. Further investigation by the publisher has found manipulation of the peer review process. The authors did not respond to requests for an explanation. As a result, the conclusions reported in the article are not considered reliable.
